# Edwin M. Fitzgerald, D.V.S.

**Published:** 1884-07

**Authors:** 


Edwin M. Fitzgerald, D.V.S., died in Greenpoint, L. I., on the 20th of April, 1884. His death was the result of accident. While driving across the track of the Long Island railroad he was struck by the express train, and instantly killed. Dr. Fitzgerald was born in White Plains, N. Y., but spent the greater part of his life in this city. He graduated from the Columbia Veterinary College in the Spring of 1882. His history as a student was exceptionally brilliant, and soon after graduating he was appointed Assistant to the Chair of Theory and Practice, a position which he filled with great credit to himself and satisfaction to his associates. He was deservedly popular as a teacher, and very successful as a practitioner. His untimely death at the early age of 28 shrouds in gloom a brilliant and promising career, and leaves an aching void in the hearts of his numerous friends and associates.

